# Complete Genome Sequence of the Quality Control Strain *Staphylococcus aureus* subsp. *aureus* ATCC 25923

**DOI:** 10.1128/genomeA.01110-14

**Published:** 2014-11-06

**Authors:** Todd J. Treangen, Rosslyn A. Maybank, Sana Enke, Mary Beth Friss, Lynn F. Diviak, David K. R. Karaolis, Sergey Koren, Brian Ondov, Adam M. Phillippy, Nicholas H. Bergman, M. J. Rosovitz

**Affiliations:** National Biodefense Analysis and Countermeasures Center, Frederick, Maryland, USA

## Abstract

*Staphylococcus aureus* subsp. *aureus* ATCC 25923 is commonly used as a control strain for susceptibility testing to antibiotics and as a quality control strain for commercial products. We present the completed genome sequence for the strain, consisting of the chromosome and a 27.5-kb plasmid.

## GENOME ANNOUNCEMENT

*Staphylococcus aureus* is an opportunistic pathogen that can cause food poisoning, as well as a variety of infections (1). *Staphylococcus aureus* subsp. *aureus* ATCC 25923 is a clinical isolate with the designation Seattle 1945 that is used as a standard laboratory testing control strain. It is sensitive to a variety of antibiotics, including methicillin. The isolate contains a staphylococcal cassette chromosome–like (SCC-like) element that lacks both recombinases and *mecA* (2, 3). We report the complete genome of the isolate, comprising a 2,778,854-bp chromosome and a 27,491-bp plasmid, pS1945.

Genomic DNA was prepared from an isolate purchased through Remel Inc. (Lenexa, KS) under license with American Type Culture Collection (ATCC), and a 10-kb insert library was sequenced on the Pacific Biosciences RSII system*. De novo* assembly with Celera Assembler version 8.1 (4) and polishing with Quiver version 0.8 (5) resulted in a single contig each for the chromosome and plasmid. The average coverage for the chromosomal contig was 408×, and average coverage for the plasmid contig was 593×.

To our knowledge, this is the first report of a native plasmid in the ATCC 25923 (Seattle 1945) strain. The plasmid was nearly identical to plasmid SAP019A from the *S. aureus* strain NRS104 (GenBank accession GQ900385), containing two small insertions relative to SAP019A.

### Nucleotide sequence accession numbers.

The complete genome sequences of the chromosome and plasmid have been deposited in DDBJ/ENA/GenBank under the accession numbers CP009361 and CP009362, respectively.
